# Supplementary material for: In situ visualization of opioid and cannabinoid drug effects using phosphosite-specific GPCR antibodies
Source: Commun Biol. 2023 Apr 15;6:419. doi: 10.1038/s42003-023-04786-2 (PMC10105690; doi:10.1038/s42003-023-04786-2)
Supplement: Supplementary file 2 — Supplementary Information [file 42003_2023_4786_MOESM2_ESM.pdf]

In situ visualization of opioid and cannabinoid drug effects using phosphosite-specific GPCR antibodies

Sebastian Fritzwanker[1], Falko Nagel[2], Andrea Kliewer[1], Viviane Stammer[1], Stefan Schulz[1,2,*]

[1]Institut für Pharmakologie und Toxikologie, Universitätsklinikum Jena, Friedrich-Schiller-Universität Jena, Drackendorfer Straße 1, D-07747 Jena, Germany

[2]7TM Antibodies GmbH, Hans-Knöll-Straße 6, D-07745 Jena, Germany

[*]Corresponding author

Email: stefan.schulz@med.uni-jena.de

Supplementary information

This .pdf includes

Supplementary Figures: 3
Supplementary Tables: 1

Supplementary Figure 1

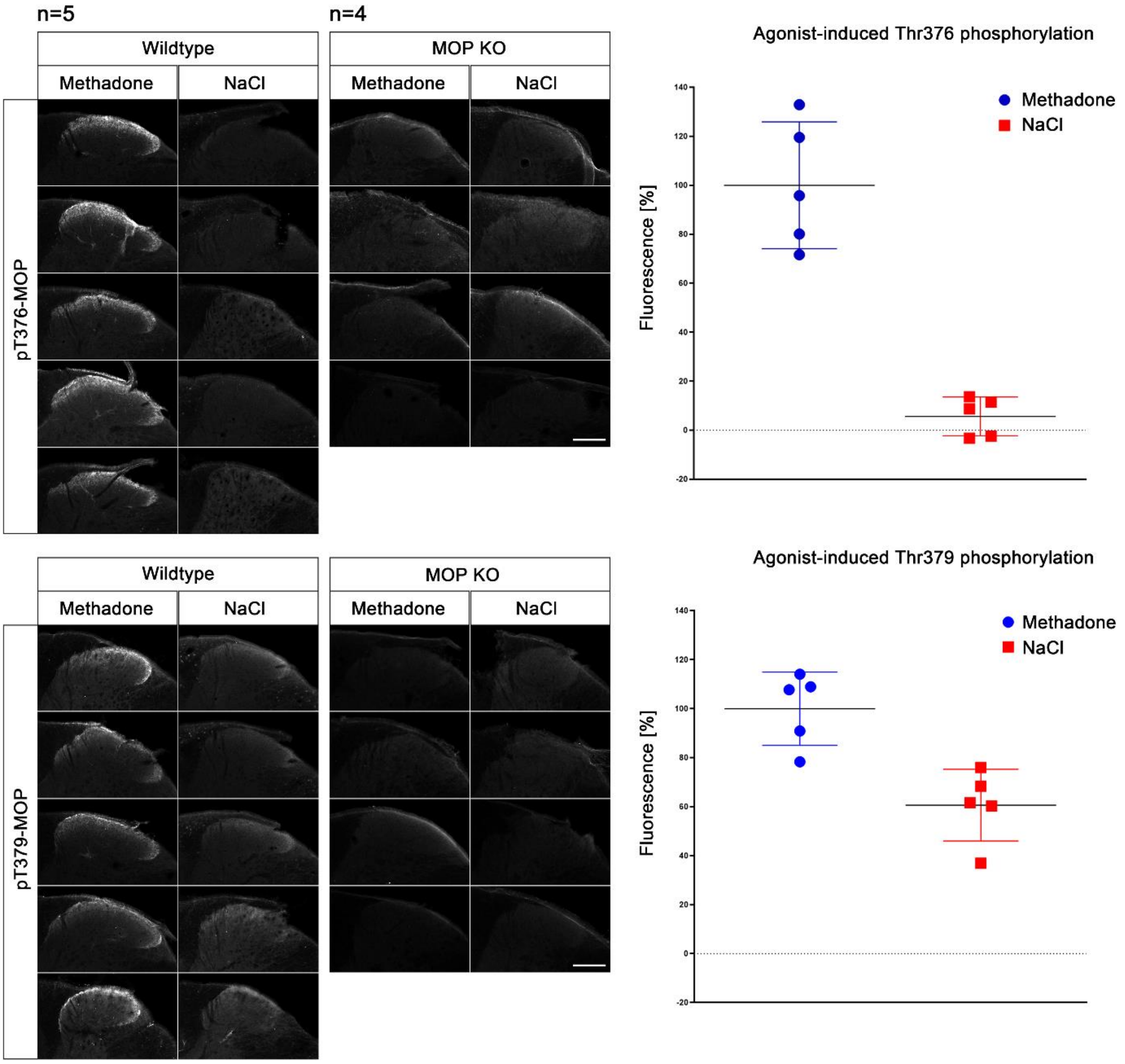

Supplementary Figure 1. Comparison of phospho-MOP staining between different mice. Animals were either treated with methadone or NaCl for 30 min, transcardially perfused, fixed and stained in the presence of PPIs. Shown are confocal images of coronal sections of the spinal cord of up to five different mice stained with either pT376-MOP or pT379-MOP antibody. MOP KO staining served as background control. Mean of five independent stained spinal cord slices is shown as 100%. Note that there is some range between the stained areas. pT379-MOP also shows a resilient phosphorylation in NaCl treated mice. ImageJ and GraphPad Prism were used for quantification. Scale bar: 250 µm.

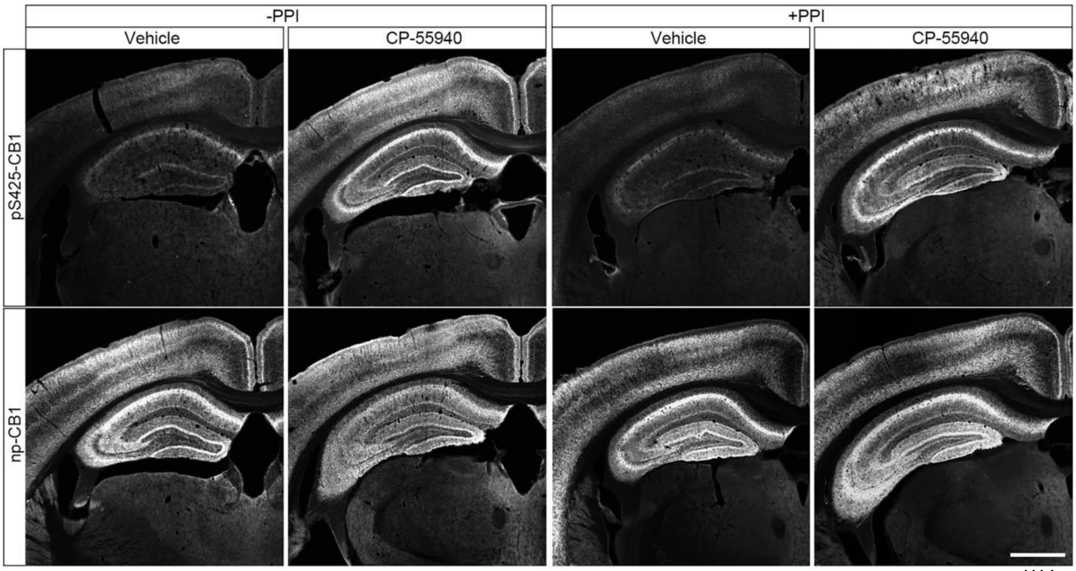

Supplementary Figure 2. Comparison of phospho-CB1 immunohistochemistry in the presence or absence of protein phosphatase inhibitors. Animals were either treated with CP-55940 or vehicle for 30 min, transcardially perfused, fixed and stained in the presence (+) or absence (-) of protein phosphatase inhibitors (PPI). Shown are confocal images of coronal sections of the brain stained with pS425-CB1 or np-CB1 antibody. Note that no PPIs are required to obtain agonist-induced phospho-CB1 immunostaining. Scale bar = 1000 µm.

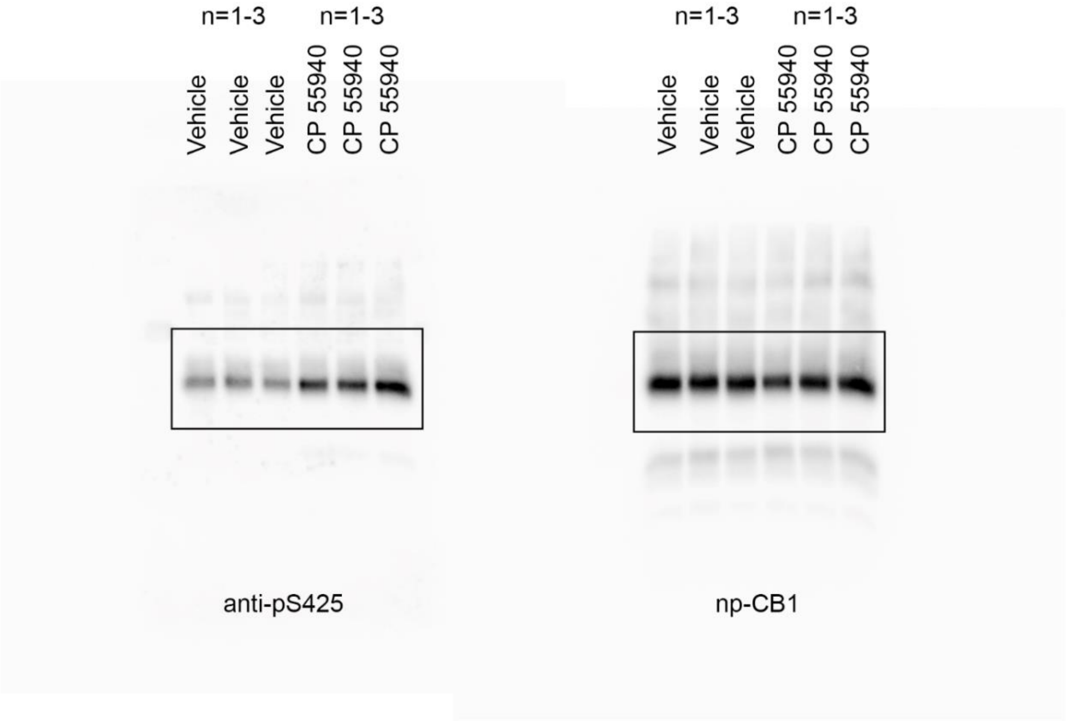

Supplementary Figure 3. Original western blot images of data represented in figure 9. The black frame marking the section displayed in the figure.

Supplementary Table 1: Source of primary antibodies.

Antibody	Catalog#	Dilution	Vendor
pT370-MOP	7TM0319B	1:500	7TM Antibodies
pS375-MOP	7TM0319C	1:100	7TM Antibodies
pT376-MOP	7TM0319D	1:400	7TM Antibodies
pT379-MOP	7TM0319E	1:400	7TM Antibodies
np-MOP	7TM0319N	1:4.000	7TM Antibodies
np-MOP	ab134054	1:500	abcam
pS425-CB1	7TM0056A	1:50	7TM Antibodies
np-CB1	1000659	1:3.500	Cayman
np-CB1	MFSR100610	1:4.000	Frontier Institute
